# Effect of acute respiratory illness on short‐term frailty status of older adults in Nakhon Phanom, Thailand—June 2015 to June 2016: A prospective matched cohort study

**DOI:** 10.1111/irv.12638

**Published:** 2019-03-07

**Authors:** Michelle M. Hughes, Prabda Praphasiri, Fatimah S. Dawood, Kanlaya Sornwong, Darunee Ditsungnoen, Joshua A. Mott, Kriengkrai Prasert

**Affiliations:** ^1^ Influenza Division Centers for Disease Control and Prevention Atlanta Georgia; ^2^ Influenza Program Thailand Ministry of Public Health ‐ U.S. Centers for Disease Control and Prevention Collaboration Nonthaburi Thailand; ^3^ Nakhon Phanom Provincial Hospital Nakhon Phanom Thailand

**Keywords:** frailty, influenza, older adults, respiratory infection, Thailand

## Abstract

**Background:**

Frailty is associated with increased risk of mortality and decline in functional status among older adults. Older adults are at increased risk of severe disease from acute respiratory illness (ARIs), but ARI effects on frailty status among older adults are not well understood. We evaluated how ARIs affect short‐term frailty status among community‐dwelling adults aged ≥65 years in Nakhon Phanom, Thailand.

**Methods:**

During May 2015 to May 2017, older adults were contacted weekly to identify ARIs as part of a community‐based longitudinal cohort study. Each participant's frailty status was assessed at baseline and every 6 months using the Vulnerable Elders Survey‐13 (VES‐13). We selected cohort participants with an ARI and compared them with a sample of participants without an ARI matched on age, sex, influenza vaccination status, and most recent VES‐13 score. For these matched cohort members, an additional VES‐13 was recorded at 3‐4 weeks after the ARI episode date.

**Results:**

Of 3220 cohort study participants, 114 participants with an ARI and 111 comparison participants without an ARI were selected for the matched cohort; three comparison participants were matched to two ARI cases. We found no statistically significant difference between ARI and non‐ARI participants in modified VES‐13 score 3‐4 weeks post‐episode (cases = 0.90, controls = 0.63, *P* = 0.07). Only two ARI episodes required hospitalization.

**Conclusions:**

Primarily mild ARIs did not affect short‐term frailty status among community‐dwelling older adults in Thailand. As few cases of severe ARI were detected, the contribution of severe ARI to changes in frailty requires further investigation.

## 1 INTRODUCTION

Globally, the burden of lower respiratory tract infections (LRTI) is highest in the youngest and oldest populations.^1^ In Thailand, there are an estimated 2 785 000 LRTI infections (defined as acute physician‐diagnosed pneumonia or bronchiolitis) annually, which contribute to an estimated 59 000 deaths.^1^ In older adults, in addition to severe outcomes such as hospitalization and death, respiratory infections may also negatively affect frailty status during and after recovery from the acute infection.^2^

Frailty has been defined in various ways but generally refers to a decline in functional status and an increased risk for adverse health outcomes, particularly in older adult populations.^3^ Acute respiratory infections may negatively impact the short‐term and long‐term frailty status of older adults. Understanding the effect of these infections on frailty status may help quantify the full impact of acute respiratory illness in older adults.

Evidence is mixed regarding the impact of acute respiratory infections of varying severity on frailty status in older adults; studies generally have used assessments of functional ability to perform activities of daily living to characterize frailty.^4^ Some studies show a decline in functional ability^2^, ^5^, ^6^ while others show no significant change post‐acute respiratory event.^7^, ^8^ The majority of these studies were conducted in high‐income countries in North America or Europe and exclusively among institutionalized adults in long‐term care facilities.

Limited data exist regarding the impact of acute respiratory infections on frailty status in non‐institutionalized, community‐dwelling older adults.^4^ In particular, there are few studies examining frailty status post‐acute respiratory infection in an Asian setting where intergenerational households are common and morbidity and mortality are high relative to other global regions.^1^, ^9^ We conducted a matched cohort study to evaluate the effect of acute respiratory illness (ARI) on the short‐term frailty status of community‐dwelling adults aged ≥65 years in Thailand.

## 2 METHODS

### 2.1 Study design

We conducted this matched cohort study within a previously described prospective longitudinal cohort study of people aged ≥65 years.^10^ People in two districts (That Phanom and Plapak) of Nakhon Phanom Province, Thailand, were enrolled into the larger cohort and followed with weekly active surveillance for episodes of ARI for 2 years. ARI was defined as a new onset of cough or worsening of chronic cough, with or without fever. Health volunteers created a master list of each person ≥65 years and performed random sampling to approach, consent, and enroll eligible community members between May 24, 2015, and July 9, 2015. At enrollment, participants completed a standardized questionnaire that included the Vulnerable Elders Survey‐13 (VES‐13), and questions about demographics, history of hospitalizations, chronic diseases and smoking, and influenza vaccination status.^10^, ^11^ Cohort participants completed subsequent VES‐13s every 6 months during the follow‐up period.

Participants who experienced an ARI episode self‐collected a nasal swab that a health volunteer picked up within 24 hours at the participant's home. During the visit, the health volunteer administered a standard questionnaire on symptoms and characteristics of the respiratory episode. If a participant was hospitalized and had a fever ≥38°C, the episode was considered a severe ARI; research nurses assigned to that health center collected a nasopharyngeal swab and administered the episode questionnaire. Swabs were transported on ice for processing, stored at −70°C, and tested at the Thailand National Institute of Health national reference laboratory using real‐time reverse transcription polymerase chain reaction (rRT‐PCR) for influenza and respiratory syncytial viruses.^12^, ^13^ The participant self‐swab method was previously validated in this population; sensitivity for detection of influenza virus was 88% and specificity was 100% compared to nasal swabs collected by trained healthcare workers.^14^ To confirm specimen quality, each was tested by rRT‐PCR for the presence of Rnase P.^14^

To measure the effect of an ARI episode on frailty status, a subset of participants who experienced their first ARI after enrollment were matched with cohort participants who had not yet experienced an ARI on age (±5 years), sex, influenza vaccination status, and most recent pre‐ARI episode VES‐13 measurement (±1 point). These matched cohort participants completed the VES‐13 survey within 3‐4 weeks after the onset of ARI in the case, which served as the primary outcome.^11^

Vulnerable Elders Survey‐13 is a questionnaire developed to screen people ≥65 years in the community to determine their risk for death or functional decline.^11^, ^15^ The questionnaire asks older adults to self‐report their age, health, limitations in physical function, and functional disabilities. The tool takes an average of <5 minutes to complete and can be administered over the phone or in person. The test has been shown to be valid and reliable.^11^, ^15^, ^16^, ^17^ For the analysis, we used a modified VES‐13 tool that excluded the age component, as age is not affected by ARI.

The three components of the VES‐13 (modified to exclude age) included health status, overall physical function, and functional disability. Health status was scored as “very good or excellent” or “good” = 0 and “fair,” “bad,” “very poor” = 1. Physical function was assessed based on self‐reported ability to perform specific tasks (ability to stoop, lift, reach, write, walk, and perform housework) using the following scale: “A lot of difficulty” or “Unable to do” = 1 vs “No difficulty,” “A little difficulty,” or “Some difficulty” = 0; if score ≥2, then 2 was the maximum value assigned. Functional disability was assessed based on self‐reported ability to perform specific activities (shop, manage money, walk across room, do light housework, and bathe) where if the person answered yes to having difficulty doing a specific activity, needed help to complete the activity, or did not do the activity because of their health, they were considered to have a disability and assigned a score of 4 and if no disability was identified, they were assigned a score of 0. The health status rating options were modified from the original VES‐13 to allow comparability to previous studies in this population.^18^ The scores for the three components were summed and could range from 0 to 7 points. Higher scores are associated with frailty and an increased risk of death or functional decline.^11^, ^15^

Sample size estimates were calculated for a matched dependent *t* test of VES‐13 scores. Assuming a mean score of 5 for ARI participants and 4 for non‐ARI participants (a higher VES‐13 score indicates increased frailty), a standard deviation of 3 for the difference between the two means, a type I error rate of 0.05, correlation of 0.2, and power of 80%, we estimated that 115 matched pairs were needed.

The Centers for Disease Control and Prevention Institutional Review Board (IRB) relied on Thailand's Ministry of Public Health, Department of Disease Control and Prevention, Ministry of Public Health Ethical Review Committee (EC) for human subjects review of the study protocol.

### 2.2 Analysis

We compared baseline characteristics between matched participants with and without an ARI exposure. We assessed the statistical significance of these differences using a paired *t* test for continuous variables, McNemar's exact test for binary variables, or the Wilcoxon signed‐rank test with continuity correction for ordinal variables. We examined characteristics of ARI episodes in cases including clinical symptoms, duration of illness, severity, laboratory testing results, and month of illness.

We reported on modified VES‐13 changes from enrollment to 18 months overall and by component score (health status, physical function, and functional disability) by ARI and non‐ARI exposure. We also examined changes between the modified VES‐13 score pre‐episode and 3‐4 weeks post‐episode. We assessed the statistical significance of baseline modified VES‐13 individual indicators between ARI and non‐ARI exposed groups using McNemar's exact test for continuous variables and Wilcoxon signed‐rank test with continuity correction for ordinal variables.

For our primary analyses, we conducted a paired *t* test comparing 3‐4 week post‐episode modified VES‐13 scores between ARI and non‐ARI participants. Because some (n = 13) of our pairs were mismatched on at least one matching criterion, we performed sensitivity analyses by excluding mismatched pairs from the analysis. We also tested for interactions between possible effect modifiers of an ARI‐frailty association including sex, age, fever during episode, and the presence of a co‐morbidity using linear mixed‐effects regression models with pairing as the random effect. Lastly, we conducted a paired *t* test on each VES‐13 category score. Statistical significance was set at *P* < 0.05. All analyses were conducted in R version 3.5.0.^19^

## 3 RESULTS

**Figure 1:**
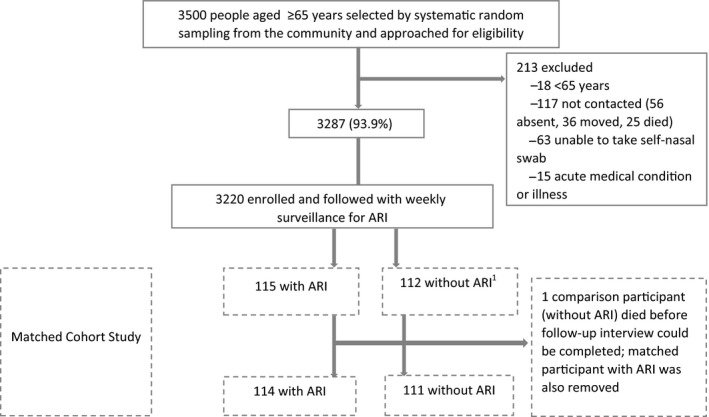
Enrollment of matched cohort study, Nakhon Phanom Province, Thailand, May 2015 to May 2017*. ^1^Three participants without acute respiratory illness (ARI) served as comparisons for two ARI cases

Of the 3500 people aged ≥65 years selected by systematic random sampling from the community and approached for eligibility, 3220 were enrolled in the cohort study between May 24 and July 9, 2015, (Figure 1) with final participant follow‐up on May 31, 2017. Overall, 115 people with ARI were identified and matched to 112 people who had not experienced an ARI in the study (three participants served as comparisons for two cases). One comparison subject died before a follow‐up interview could be completed, so this matched pair was not analyzed. The final matched cohort sample included 114 ARI matched to 111 non‐ARI participants.

Baseline sociodemographic and health characteristics were similar between ARI and non‐ARI participants with the exception of number of household members, income, and education level (Table 1). Mean modified VES‐13 scores were low overall (1.1 for ARI cases and 1.2 for non‐ARI participants). Individual modified VES‐13 components were similar between those with and without ARI (Table 2). ARI episode dates ranged from March 11, 2016, through June 21, 2016. Nasal congestion, sore throat, and fever were each reported in over half the episodes (Table 3). Only two severe ARI episodes (2%) were identified. Median illness duration was 6 days. Influenza was detected in 3% (n = 3) of episodes.

**Table 1:** Comparison of participant characteristics at baseline enrollment, Nakhon Phanom Province, Thailand, May 2015 to May 2017

	Matched cohort without ARI exposure n = 114^a^		Matched cohort with ARI exposure n = 114		Total cohort^b^ N = 3220	
Demographics						
Age (mean, SD)	72.4	4.8	72.9	4.8	72.7	5.3
Female sex	72	63%	72	63%	1896	59%
Married	57	50%	55	48%	1730	54%
Number of household members^c^ (mean, SD)	4.0	2.2	3.3	1.7	3.7	1.9
That Phanom District resident	57	50%	56	49%	2066	64%
Low income^c^, ^d^	60	53%	84	74%	1895	59%
Highest education^c^						
Never attended school	5	4%	15	13%	226	7%
Primary school	99	87%	93	82%	2773	86%
Secondary school and over	10	9%	6	5%	216	7%
Health status						
Vaccinated 2015‐16 season^e^	62	54%	61	54%	1666	52%
Vaccinated 2016‐17 season^f^	57	50%	51	45%	1499	47%
Matching vaccinated status^g^	59	52%	57	50%	NA	NA
≥1 hospitalization in past year	18	16%	18	16%	574	18%
VES‐13						
Modified VES‐13 score at enrollment^h^ (mean, SD)	1.12	1.52	1.20	1.76	1.39	2.04
Most recent modified VES‐13 score prior to cases’ illness episodes^i^ (mean, SD)	0.86	1.47	0.99	1.75	NA	NA
Smoking						
Current smoker	22	19%	15	13%	533	17%
Underlying medical conditions						
≥1 underlying medical condition	42	37%	48	42%	1166	36%
Chronic heart and circulatory disease	32	28%	32	28%	822	26%
Metabolic disease	12	11%	18	16%	457	14%
Chronic lung disease	3	3%	4	4%	117	4%
Chronic kidney disease	4	4%	2	2%	85	3%
Other health condition^j^	5	4%	3	3%	82	3%

a Three matched cohort participants without ARI exposure served as a control twice (111 unique participants without ARI).

b Participants from entire study cohort from which the matched cohort population was selected.

c Participants with and without an ARI episode were statistically significantly different in number of household members, income level, and education assessed by paired *t* tests, McNemar's exact tests, or the Wilcoxon signed‐rank tests with continuity correction.

d Low income defined as monthly income <5000 Baht. In Thailand, national incomes <7368 Baht were categorized as low to moderate income^24^; <5000 Baht was the closest income to limit to this benchmark.

e Vaccinated for the 2015‐16 influenza season defined as June 2015 to May 2016; Vaccination occurred during May to September 2015.

f Vaccinated for the 2016‐17 influenza season defined as June 2016 to May 2017; Vaccination occurred during May to September 2016.

g Vaccinated for season where episode occurred: 2015‐16 influenza season vaccine for episodes during June 2015 to May 2016; 2016‐17 influenza season vaccine for episodes during June 2016 to May 2017. Ten pairs (9%) were mismatched on vaccination status.

h Frailty status assessed using the modified Vulnerable Elders Survey (VES‐13) at cohort enrollment. A higher score correlates with increased frailty.

i Most recent frailty status is one assessed prior to ARI event using the modified VES‐13 prior to enrollment in the matched cohort study. Four pairs (4%) were mismatched on modified VES‐13 score.

j Includes cerebrovascular disease (stroke), chronic liver disease, neurologic/neuromuscular disorder, hemoglobinopathy, immunosuppressive condition, lupus, or other cancer.

**Table 2:** Comparison of baseline modified VES‐13 measures between non‐ARI and ARI matched cohort, Nakhon Phanom Province, Thailand, May 2015 to May 2017

	No ARI exposure n = 114^a^ (%)		ARI exposure n = 114 (%)	
Health status				
**Very good or excellent or good**	71	62	75	66
**Fair, bad, or very poor**	43	38	39	34
Physical function^b^				
Stooping, crouching, or kneeling^c^	7	6	1	1
Lifting or carrying objects = 5 k	7	6	4	4
Reaching or extending arms above shoulder level	1	1	0	0
Writing or handling and grasping small objects	1	1	0	0
Walking 0.5 km	8	7	10	9
Heavy housework	4	4	4	4
**Overall** d **—Low**	6	5	5	4
**Overall—Medium**	12	11	7	6
**Overall—High**	96	84	102	90
Functional disabilities^e^				
Shopping for personal items	4	4	9	8
Managing money	4	4	6	5
Walking across the room	2	2	3	3
Light housework	3	3	3	3
Bathing or showering	0	0	1	1
**≥1 functional disability**	6	5	11	10

a Three matched cohort participants without ARI exposure served as a control twice (111 unique participants without ARI).

b Difficulty in performing specific tasks (“A lot of difficulty” or “Unable to do” vs “No difficulty,” “A little difficulty,” or “Some difficulty”).

c There were no statistically significant differences in individual VES‐13 indicators between participants with and without an ARI episode as assessed by McNemar's exact tests and Wilcoxon signed‐rank tests with continuity correction.

d Overall physical function category score (≥2 = Low, 1 = Medium, 0 = High).

e Because of your health or physical condition do you have any difficulty doing a specific activity and get help to complete activity? If you do not do activity, is it because of your health? An answer of “yes” to either of these questions was considered being positive for having that particular functional disability.

**Table 3:** Characterization of ARI episodes in matched cohort, Nakhon Phanom Province, Thailand, May 2015 to May 2017, N = 114

	All episodes n = 114	
Clinical symptoms		
Cough	114	100%
Nasal congestion	78	68%
Sore throat	76	67%
Fever	63	55%
Severe ARI^a^	2	2%
Illness duration (median, IQR)	6	(4‐8)
Laboratory PCR result		
Influenza positive	3	3%
RSV positive	0	0%
Time from symptom onset to specimen collection (median, IQR)	2	(2‐3)
Month		
March	25	22%
April	41	36%
May	27	24%
June	21	18%

a Severe ARI defined as new onset of cough, or worsening of chronic cough with a fever ≥38.0°C that required hospitalization.

We found no statistically significant difference between cases and controls in their post‐episode modified VES‐13 score (cases = 0.90, controls = 0.63, *P* = 0.07). A subset of cases were mismatched on vaccination status (n = 9) or modified VES‐13 score (n = 3) or both (n = 1). To assess the sensitivity of our results to these mismatches in the design phase, we performed statistical tests excluding these matched pairs and found no differences in our results. We also fit linear mixed‐effects regression models with interaction terms for sex, age, the presence of a co‐morbidity, or fever present in episode and found no evidence of interaction with any of these variables. When broken down by VES‐13 component, we found no statistically significant differences between cases and controls for health, activity, or function. Mean and individual differences in modified VES‐13 scores pre‐ and post‐ARI were visualized to look for trends (Figures S1 and S2). Mean modified VES‐13 scores from the four recorded time points were also visualized to examine trends over the entire study period (Figure S3).

## 4 DISCUSSION

We found no difference in modified VES‐13 scores at 3‐4 weeks post‐episode between older adults who did and did not experience an acute respiratory event. When modified VES‐13 component (health status, physical function, and functional disability) scores were examined, there were also no differences between ARI and non‐ARI participants and relatively little change in scores over the entire 18‐month study period.

One reason for our findings may be that our sample at baseline was on average non‐frail (mean modified VES‐13 score <2) relative to the sample we had planned to capture. Thus, they may have been less susceptible to the negative outcomes of respiratory infection compared to older adults with higher baseline frailty. Previous studies using the VES‐13 scale were in frail populations where baseline VES‐13 scores were substantially higher than in our study.^15^, ^16^ For example, Saliba et al^11^ found that >30% of sampled Medicare beneficiaries had a VES‐13 ≥3, compared to only 11% in our study, indicating a less frail population in our Thai cohort compared to the United States. Further, the VES‐13 was developed in the United States and may not work well to capture frailty in a Thai population.^11^ Moreover, our study population may be healthier than the general older adult population in Thailand as assessed by the percentage who self‐reported good or very good overall health.^20^, ^21^

The ARIs we identified tended to be mild. Specifically, almost half of the infections did not include fever as part of the illness and only two of the episodes were classified as severe ARI. Higher severity respiratory episodes may have had a more pronounced detrimental impact on frailty compared to lower severity episodes. For some cases with chronic respiratory disease, the episodes may have only been an exacerbation of their underlying condition. All episodes occurred between March and June, which had little overlap with peak influenza and respiratory syncytial virus (RSV) season in Thailand.^22^, ^23^ Laboratory findings reflected this seasonality with no RSV detection and 3% positivity for influenza. If episodes had been enrolled during peak periods of influenza/RSV circulation, we may have captured more severe disease. We also did not examine longer term effects of acute respiratory illness on frailty status, although the mean change among both ARI and non‐ARI participants across the 18‐month study period was small.

There were several limitations in this study. First, 12% of ARI and non‐ARI participants were mismatched on at least one matching criterion. This reduced the power of our study to detect differences when restricting the analysis to non‐mismatched pairs. Second, we were not powered to detect a mean difference in modified VES‐13 score of <1 between ARI and non‐ARI participants, although it is unlikely that smaller differences in modified VES‐13 score would be meaningful. Third, we identified predominantly mild ARI episodes which limited our ability to evaluate the impact of severe ARI episodes on frailty status. Lastly, our study population was in relatively good health with low VES‐13 scores, so were limited in understanding whether an ARI leads to increased frailty among those with higher baseline modified VES‐13 who are likely most vulnerable to poor outcomes after ARI.

This study was a representative, population‐based community study that examined the association between acute respiratory infections and one measurement of subsequent frailty in older adults in Thailand. This was one of the first studies to examine this association among community‐dwelling older adults. While we found no association in our study population, additional research is needed to examine the impact of severe acute respiratory illnesses among older adults, especially those who are frailer at baseline.

## DISCLAIMER

The findings and conclusions in this report are those of the authors and do not necessarily represent the official position of the Centers for Disease Control and Prevention.
