# Supplementary material for: Effect of acute respiratory illness on short‐term frailty status of older adults in Nakhon Phanom, Thailand—June 2015 to June 2016: A prospective matched cohort study
Source: Influenza Other Respir Viruses. 2019 Mar 7;13(4):391–7. doi: 10.1111/irv.12638 (PMC6586187; doi:10.1111/irv.12638)
Supplement: Supplementary file 1 [file IRV-13-391-s001.docx]

## Supplemental Figure 1: Modified VES-13 Individual Changes from Pre-Episode to Post-Episode for ARI and Non-ARI Exposed Matched Cohort, Nakhon Phanom Province, Thailand, May 2015–May 2017^1^


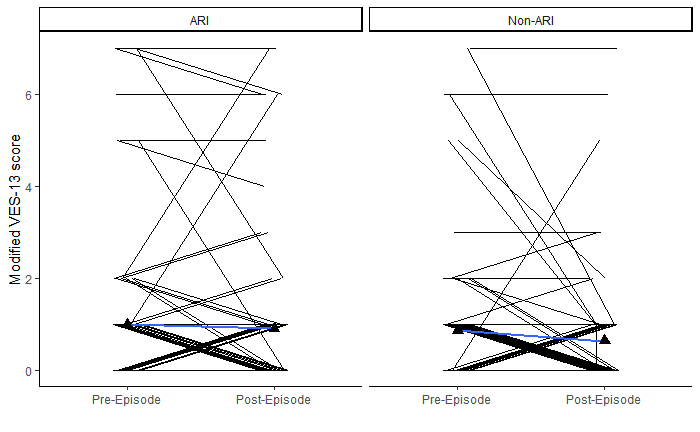


^1^ Blue line represents mean change from pre-episode to post-episode time points.
