# Supplementary material for: Effect of acute respiratory illness on short‐term frailty status of older adults in Nakhon Phanom, Thailand—June 2015 to June 2016: A prospective matched cohort study
Source: Influenza Other Respir Viruses. 2019 Mar 7;13(4):391–7. doi: 10.1111/irv.12638 (PMC6586187; doi:10.1111/irv.12638)
Supplement: Supplementary file 2 [file IRV-13-391-s002.docx]

## Supplemental Figure 2: Modified VES-13 Mean Changes from Pre-Episode to Post-Episode for ARI and Non-ARI Exposed Matched Cohort, Nakhon Phanom Province, Thailand, May 2015–May 2017^1^

|  | \|  \| \| --- \| |  |  |  |  |  |
| --- | --- | --- | --- | --- | --- | --- | --- |
|  |  |  |  |  |  |  |
|  |  |  |  |  |  |  |
|  |  |  |  |  |  |  |
|  |  |  |  |  |  |  |
|  |  |  |  |  |  |  |
|  |  |  |  |  |  |  |
|  |  |  |  |  |  |  |

^1^ Modified VES-13 composite score range = 0–7. Individual VES-13 components value ranges: Health Status (0,1), Physical Function (0,1,2), and Functional Disability (0,4).
